# Effect of Extrusion Processing on the Instant Properties and Structural Characteristics of Yellow Waxy Corn Flour

**DOI:** 10.3390/foods15172974

**Published:** 2026-08-25

**Authors:** Hong Guo, Zhongdong Zhang, Zhe Cheng, Qi Li, Yunlong Li

**Affiliations:** 1 Shanxi Institute for Functional Food, Shanxi Agricultural University, Taiyuan 030031, China; guohong@sxau.edu.cn (H.G.);; 2 Maize Research Institute, Shanxi Agricultural University, Xinzhou 034000, China

**Keywords:** yellow waxy corn, extrusion cooking, reconstitution property, caking rate, microstructure, physicochemical characteristics

## Abstract

Instant cereal powders often suffer from poor reconstitution and caking during preparation. This study investigated the effects of twin-screw extrusion and particle-size regulation on the reconstitution properties and structural characteristics of yellow waxy corn flour. Nine extrusion treatments (140–180 °C; 12–18% feed moisture) and four particle-size fractions (40–80 mesh) were screened using caking rate and suspension stability as evaluation indices; no formal process optimization was performed. Three 60-mesh samples produced at 180 °C with different feed moisture levels were then selected as representative treatments for mechanistic characterization. Compared with native flour, the extruded samples showed lower caking, improved suspension stability, and substantially higher water absorption. SEM and DSC analyses demonstrated conversion of intact granules into a porous matrix and a marked decrease in residual gelatinization enthalpy, whereas XRD showed retention of the A-type diffraction pattern. FTIR spectra were broadly similar among samples and did not indicate formation of new major functional groups. These results connect extrusion-induced granular and thermal changes with improved hydration and provide a basis for developing clean-label instant waxy corn products without chemical anti-caking agents.

## 1. Introduction

With continuous improvements in living standards and growing public focus on dietary health, modern consumers have raised stricter requirements for the nutritional value, processing convenience and additive safety of staple and auxiliary food products [1]. Waxy corn is a distinctive minor grain crop native to China, which is rich in high-branched amylopectin, dietary fiber, multiple vitamins and mineral elements. It possesses a soft, glutinous taste and comprehensive nutritional advantages, making it widely favored by consumers across all age groups [2,3]. Nevertheless, the industrial processing chain of waxy corn still faces prominent bottlenecks at present. Commercial waxy corn products are dominated by quick-frozen fresh ears and vacuum-preserved grains, featuring single product forms, insufficient deep processing depth, low additional economic value and outdated supporting production lines. These limitations severely restrict the industrialization and value-added development of waxy corn resources. Accordingly, exploring novel deep-processing technologies and developing high-value instant grain products has become an urgent research direction for cereal food researchers.

Extrusion cooking, superfine pulverization, and microwave thermal treatment are commonly used to produce instant cereal powders [4]. Twin-screw extrusion subjects cereal materials to simultaneous heat, pressure, and shear [5], which can gelatinize starch, alter protein conformation, fragment macromolecules, and generate porous extrudates [6,7]. However, prior extrusion studies have largely emphasized expansion, digestibility, or conventional physicochemical properties, frequently using isolated starches or snack-type products. The evidence is more limited for whole waxy corn flour intended for direct hot-water reconstitution, particularly regarding (i) how barrel temperature and feed moisture jointly affect caking and suspension behavior, (ii) how post-extrusion particle size modifies that behavior, and (iii) whether granular, crystalline, thermal, and hydration characteristics collectively explain the observed reconstitution response. Consequently, a direct process–structure–reconstitution relationship for instant yellow waxy corn flour remains insufficiently defined.

Commercially available packaged instant grain powders generally suffer from severe particle agglomeration when mixed with hot water, which seriously damages edible mouthfeel and consumer experience [8]. To solve the caking defect, food manufacturers commonly add synthetic anti-caking agents during production [9]. Nevertheless, these chemical additives carry potential hidden dangers to human long-term health. Non-dairy creamer, a widely used auxiliary material in instant powder products, mainly consists of hydrogenated vegetable oil containing trans fatty acids, which are difficult for the human digestive system to metabolize and decompose. Long-term excessive intake will raise the risk of obesity, hyperlipidemia and cardiovascular diseases [10]. Therefore, revealing how physical extrusion treatment drives spontaneous structural transformation of intrinsic starch and protein molecules to optimize hydration performance without exogenous chemical additives has become a hot research topic in the field of clean-label instant cereals.

In the present work, yellow waxy corn flour was processed using twin-screw extrusion followed by particle-size grading. A 3 × 3 treatment matrix combining three barrel temperatures and three feed moisture levels was used for exploratory screening rather than formal optimization. Caking rate and suspension stability were first evaluated across the treatments and particle-size fractions. The 60-mesh fraction was used for subsequent comparisons, and three samples produced at 180 °C with 12%, 15%, and 18% feed moisture were selected to provide a common high-temperature background with measurable variation among the three feed-moisture treatments in reconstitution behavior. Nutritional composition, SEM, particle-size distribution, XRD, FTIR, DSC, water absorption, rheology, and color were then evaluated to relate structural changes to powder performance. The study therefore advances current knowledge by linking process variables and particle-size regulation with hot-water caking and suspension behavior in whole yellow waxy corn flour, while distinguishing screening-based sample selection from statistical process optimization.

## 2. Materials and Methods

### 2.1. Materials

Yellow waxy corn harvested in 2024 was obtained from Qinhuangdao, Hebei Province, China. After manual dehulling and degerming, the endosperm was milled and passed through a 100-mesh sieve to obtain native yellow waxy corn flour for subsequent experiments. All chemicals and reagents used in this study were of analytical grade.

### 2.2. Extrusion Processing and Sample Preparation

To generate samples with different degrees of thermomechanical modification, three barrel temperatures (140, 160, and 180 °C) and three feed moisture levels (12%, 15%, and 18%) were cross-combined to form nine treatments. This 3 × 3 design was used for comparative screening and sample generation. No response-surface methodology, desirability function, regression-based optimum, or validation of a predicted optimum was performed; therefore, the terms ‘optimized condition’ and ‘optimized sample’ are not used to describe the selected treatments in this study.

Pretreated yellow waxy corn flour with adjusted moisture was fed into an FWHE22-32 high-moisture co-rotating twin-screw extrusion system (Hunan Fumach Food Engineering Technology Co., Ltd., Changsha, China). The extruder was equipped with modular twin screws and a single-hole circular outlet die with a die-hole diameter of 5 mm. The screw speed was 260 rpm, and the feed rate was 11.66 kg/h. The detailed screw-element arrangement was not retained in the experimental records and was therefore unavailable. After extrusion and expansion, the fresh extrudates were dried at 45 °C to constant weight, pulverized, and sequentially sieved through 40–80 mesh standard sieves. The resulting fractions were evaluated for caking and phase separation to select representative samples for subsequent structural and rheological analyses.

### 2.3. Determination of Suspension Stability and Caking Rate

The phase-separation behavior and caking rate of reconstituted yellow waxy corn flour were determined according to Liu et al. with slight modifications [11]. Briefly, 5.0 g of sample was dispersed in 50 mL of deionized water preheated to 80 °C and stirred for 30 s. After standing for 5 min, the height of the clear supernatant layer (h_1_) and the total height of the liquid column (h) were recorded. The supernatant separation ratio (K) was calculated using Equation (1).

(1) $$K=\frac{h_{1}}{h}\times 100\%$$

where h_1_ is the height of the clear supernatant layer (cm), and h is the total height of the liquid column (cm). *K* is expressed as a percentage; a lower K value indicates less phase separation and therefore greater suspension stability.

Subsequently, the suspension was filtered through a pre-weighed 20-mesh sieve. The retained agglomerates were rinsed with deionized water and dried to a constant weight. The caking rate (*Q*) was calculated according to Equation (2).

(2) $$Q=\frac{m_{2}-m_{1}}{m}\times 100\%$$

where *m*_1_ is the initial mass of the sieve (g), *m*_2_ is the mass of the sieve with dried agglomerates (g), and *m* is the mass of the sample (5.0 g).

All measurements were performed in triplicate.

### 2.4. Proximate Nutritional Analysis

The total starch, reducing sugar, and total protein contents were determined according to GB 5009.9-2023, GB 5009.7-2016, and GB 5009.5-2016, respectively [12,13,14]. Total protein was determined from total nitrogen using the conversion procedure specified in GB 5009.5-2016 [14]. All measurements were performed in triplicate.

### 2.5. Laser Particle Size Distribution

The particle size distribution and specific surface area (SSA) of native and extruded yellow waxy corn flour were determined using a laser particle size analyzer (BT-2001, Bettersize Instruments Ltd., Dandong, China) according to the method described by Ali and Hasnain (2014) with slight modifications [15]. Briefly, the powder samples were dispersed in absolute ethanol at a solid-to-liquid ratio of 1:300 (*w*/*v*). The suspension temperature was maintained at 20 ± 0.5 °C, and the laser obscuration range was controlled between 10% and 20%. The median particle diameter (D_50_) and SSA were recorded, and each sample was analyzed at least three times.

### 2.6. Scanning Electron Microscopy (SEM)

The surface morphology of native and extruded yellow waxy corn flour was observed using a field emission scanning electron microscope (JSM-7001F, JEOL Ltd., Tokyo, Japan) according to the method described by Arribas et al. (2019) with slight modifications [16]. Briefly, the samples were fixed on a conductive adhesive tape and coated with gold under vacuum conditions (15 mA, 5 Pa, 80 s). The microstructure of the samples was observed and captured at an accelerating voltage of 5.0 kV and magnifications of 1000× and 2000×.

### 2.7. Fourier-Transform Infrared Spectroscopy (FTIR)

The molecular structural changes of native and extruded yellow waxy corn flour were analyzed using Fourier-transform infrared spectroscopy (FTIR) (Nicolet iS20, Thermo Fisher Scientific, Instruments Co., Ltd., Shanghai, China) according to the method described by Sivam et al. (2013) with slight modifications [17]. Briefly, the samples were thoroughly mixed with potassium bromide (KBr) at a mass ratio of 1:100 and pressed into pellets. The spectra were collected in the range of 4000–400 cm^−1^ with 64 scans at a resolution of 4 cm^−1^.

### 2.8. X-Ray Diffraction (XRD)

The crystalline structure changes of native and extruded yellow waxy corn flour were determined using an X-ray diffractometer (D/MAX-2600, Rigaku Corporation, Tokyo, Japan) according to the method described by Liu et al. (2021) with slight modifications [18]. The samples were scanned over a diffraction angle (2θ) range of 5–40° at a voltage of 40 kV and a current of 200 mA, with a step size of 0.02° and a scanning speed of 2°/min. The relative crystallinity was calculated based on the integrated areas of crystalline and amorphous regions.

### 2.9. Differential Scanning Calorimetry (DSC)

The thermal transition characteristics of native and extruded yellow waxy corn flour were determined using differential scanning calorimetry (DSC200, HITACHI, Tokyo, Japan) according to the method described by Torbica et al. (2021) with slight modifications [19]. Briefly, approximately 3 mg of sample was weighed into an aluminum pan, mixed with deionized water at a solid-to-water ratio of 3:7 (*w*/*w*), and sealed for equilibration at room temperature for 12 h. The samples were heated from 25 to 200 °C at a heating rate of 10 °C/min under a nitrogen atmosphere (30 mL/min). The thermograms were recorded, and the gelatinization parameters, including onset temperature (T_o_), peak temperature (T_p_), conclusion temperature (T_c_), and gelatinization enthalpy (ΔH), were analyzed.

### 2.10. Color Measurement

The color characteristics of native and extruded yellow waxy corn flour were determined using a colorimeter (CS-580A, Shofound Electronics Co., Ltd., Shanghai, China) according to the method described by Oliveira et al. (2017) with slight modifications [20]. The color parameters, including lightness (L*), redness/greenness (a*), and yellowness/blueness (b*), were measured for raw flour and extruded samples. Each sample was measured three times, and the average values were recorded.

### 2.11. Water Absorption Index (WAI)

The water absorption index (WAI) of native and extruded yellow waxy corn flour was determined according to the method described by Yu et al. (2017) with slight modifications [21]. Briefly, 1.0 g of sample was dispersed in 20 mL of warm water (30 °C) and maintained in a water bath at 30 °C for 30 min with continuous stirring. The suspension was then centrifuged at 3000 rpm for 15 min, and the mass of the precipitated fraction was recorded. Each sample was analyzed in triplicate, and the WAI value was calculated according to the following Equation (3):

(3) $$WAI(g/g)=\frac{m_{1}}{m}$$

where *m*_1_ represents the mass of the sediment (g), and *m* represents the mass of the sample (g).

### 2.12. Rheological Analysis

The rheological properties of reconstituted yellow waxy corn porridge were determined using a rotational rheometer (MCR 302, Anton Paar, Graz, Austria) equipped with a 40 mm parallel-plate geometry with a gap of 2 mm. The measurements were performed according to the method described by Liu et al. with slight modifications [22]. Briefly, the flour samples were mixed with deionized water at a ratio of 1:9 (*w*/*w*) to obtain a homogeneous porridge. The samples were equilibrated at 25 °C before analysis. Dynamic rheological measurements were carried out within the linear viscoelastic region at a constant strain of 0.5% and a frequency sweep ranging from 0.1 to 100 rad/s. The storage modulus (G′), loss modulus (G″), and loss tangent (tan δ = G″/G′) were recorded. All measurements were performed in triplicate.

### 2.13. Statistical Analysis

All experimental data were expressed as mean ± standard deviation (SD). One-way analysis of variance (ANOVA) and Duncan’s multiple range test were performed by SPSS 27.0 software to evaluate significant differences between groups (*p* < 0.05). Origin 2025 software was used for data sorting, fitting and graph drawing.

## 3. Results and Discussion

### 3.1. Screening of Extrusion Treatments Based on Reconstitution Properties

Particle-size screening was conducted before comparing the extrusion treatments because particle size directly affects wetting, interparticle contact, and sedimentation. Across the 40–80 mesh fractions (Tables S1 and S2), coarse powders showed less caking but poorer suspension behavior, whereas very fine powders suspended more readily but tended to agglomerate because of their greater external surface area. The 60-mesh fraction provided the most suitable compromise between these two responses and was therefore fixed for the subsequent treatment comparison and structural analyses. Accordingly, Table 1 and Table 2 present the 60-mesh data, while the complete particle-size results are provided in Tables S1 and S2.

As shown in Table 1, Q differed substantially among the nine tested treatment groups. At 140 °C, Q was relatively high at feed moisture levels of 15% and 18% (2.66% and 1.20%, respectively), whereas the value at 12% moisture was lower (0.40%). At 160 °C, all three treatments exhibited uniformly low Q values (0.14–0.16%). At 180 °C, Q also remained generally low (0.13–0.60%), although YC-T180M12 showed a higher value than YC-T160M12. Therefore, increasing temperature did not result in a uniform reduction in caking across all tested groups. The observed differences may reflect competing effects of starch gelatinization, molecular fragmentation, and shear intensity on particle wetting and agglomerate formation. In particular, a low-moisture, high-shear environment may promote matrix disruption but may also favor rapid surface hydration and the formation of poorly dispersible agglomerate shells. These mechanisms were not measured directly and require confirmation through measurements such as specific mechanical energy, degree of gelatinization, and wetting kinetics. This treatment-dependent response is consistent with recent extrusion studies showing that flour hydration and reconstitution properties are governed by the balance among gelatinization, molecular fragmentation, and moisture-related changes in shear intensity [11].

The supernatant separation ratio (Table 2) also varied among the tested treatment groups. K ranged from 8.00% to 20.00% at 140 °C, from 8.00% to 11.00% at 160 °C, and from 4.00% to 11.00% at 180 °C. Because K represents the relative height of the clear supernatant layer, lower values indicate less phase separation and better suspension stability. YC-T180M15 therefore exhibited the least separation, whereas YC-T180M18 did not improve relative to the corresponding 160 °C treatment. Thus, K did not change uniformly with increasing temperature or feed moisture across all treatment groups. This variation is consistent with reports that feed moisture may facilitate starch plasticization while simultaneously reducing mechanical shear, resulting in treatment-specific changes in hydration and suspension behavior rather than a universal linear response [21].

The particle-size results also explain why treatment selection could not be based on Q alone. Fine particles provide faster suspension but create more particle–particle contact area and can form cohesive agglomerates, whereas coarse porous particles wet more slowly and sediment more readily. Fixing the fraction at 60 mesh therefore reduced particle-size variability and allowed the effects of the extrusion treatments to be compared on a common basis.

**Figure 1:**
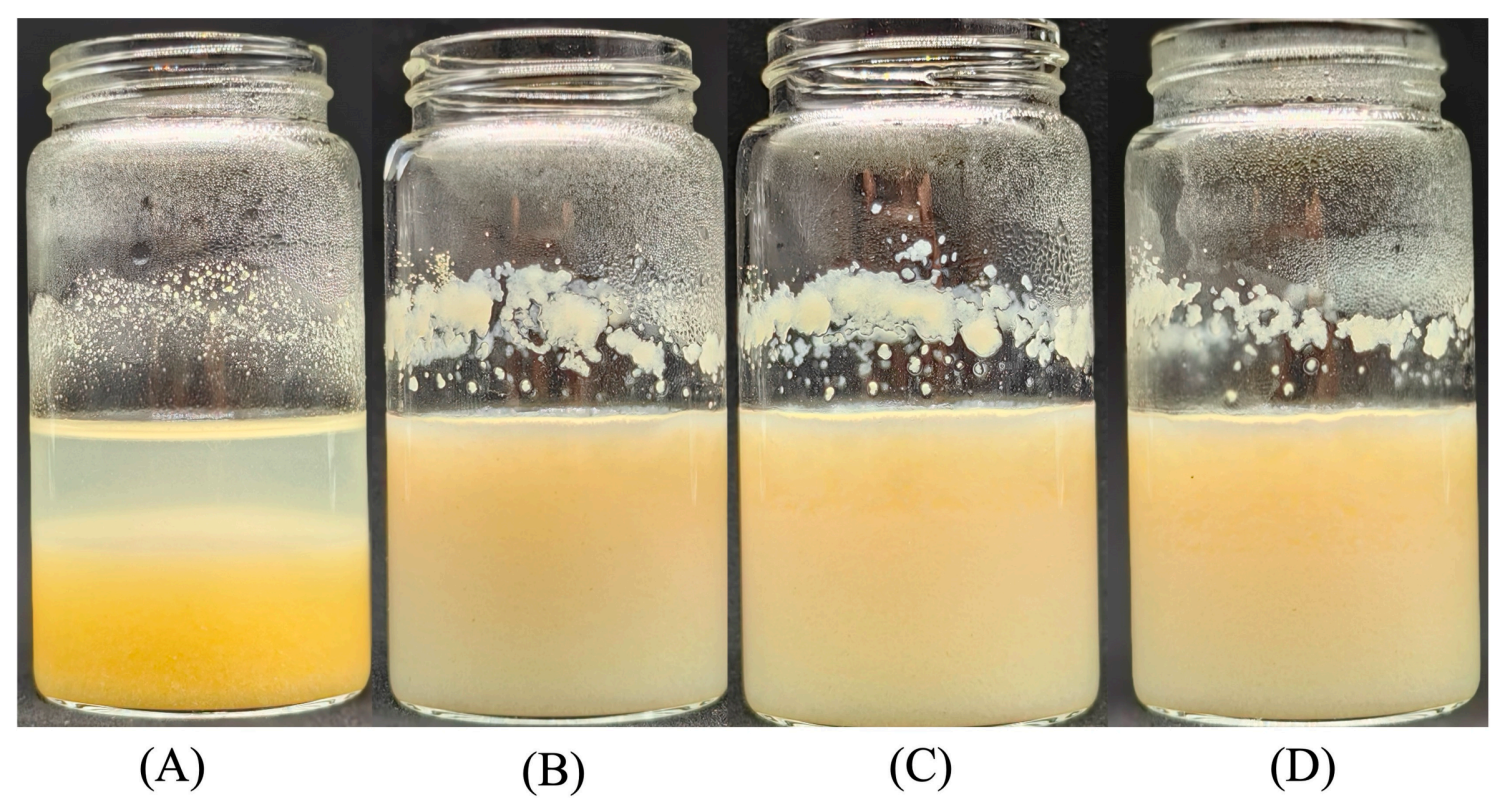
Representative photographs of the reconstituted yellow waxy corn flour samples after dispersion in hot water and standing for 5 min. (**A**) YC-YL; (**B**) YC-T180M12; (**C**) YC-T180M15; (**D**) YC-T180M18.

Representative photographs of the reconstituted yellow waxy corn flour samples are shown in Figure 1. Compared with the native flour (YC-YL), the extruded samples exhibited a more homogeneous appearance with reduced sedimentation after standing, visually confirming the improved reconstitution behavior achieved by extrusion. Among the extruded samples, YC-T180M15 and YC-T180M18 maintained relatively uniform dispersions, which were generally consistent with the measured caking rate and suspension stability.

Based on this screening, YC-T180M12, YC-T180M15, and YC-T180M18 were selected as representative, not optimized, samples for further investigation, with native flour (YC-YL) as the control. The 180 °C series was chosen for three reasons: it maintained generally low caking across all moisture levels; it provided a common high-temperature background expected to produce clear thermomechanical modification relative to native flour; and, unlike the nearly invariant Q values at 160 °C, it retained measurable variation among the three feed-moisture treatments in both Q (0.13–0.60%) and K (4.00–11.00%). This selection enabled the effect of feed moisture on structure and hydration to be examined at a fixed temperature. It does not establish 180 °C as a global optimum, because the study did not use a formal optimization model or validate a predicted optimum.

### 3.2. Structural Evolution of Representative Extruded Flour Samples

#### 3.2.1. Effect of Extrusion Modification on Nutritional Composition of Waxy Corn Flour

Thermomechanical shear during extrusion induces macromolecular rearrangement in starch and protein, thereby modulating the nutritional composition of waxy corn flour [23]. As presented in Table 3, native YC-YL contained 73.0 ± 0.5% total starch. After extrusion at 180 °C, the total starch content slightly rose from 73.1% for YC-T180M12 to 75.3 ± 0.7% for YC-T180M18 with increasing feed moisture. Mechanical fragmentation of intact starch granules liberates starch trapped within dense granular matrices, contributing to higher quantified starch yields after extrusion treatment.

Reducing sugars were measured at 1.0 ± 0.0% in untreated YC-YL but were below the detection limit in YC-T180M12, YC-T180M15, and YC-T180M18. Their apparent loss may have resulted from several processes during extrusion, including participation in Maillard reactions, caramelization, thermal degradation, or changes in extractability. However, Maillard-specific products, browning intermediates, and volatile compounds were not measured in this study. Therefore, the present data show only a decrease below the analytical detection limit and do not experimentally confirm Maillard condensation or aroma formation.

The values reported in Table 3 represent total protein determined according to GB 5009.5-2016 [14,24], rather than soluble protein. The measured total protein content increased from 0.10 ± 0.00% in YC-YL to 0.56 ± 0.02%, 0.63 ± 0.03%, and 0.66 ± 0.01% in YC-T180M12, YC-T180M15, and YC-T180M18, respectively (*p* < 0.05). Because the method quantifies total nitrogen-derived protein, these results should not be interpreted as evidence of increased protein solubility or protein unfolding. The apparent differences may reflect changes in dry-matter composition or analytical recovery and require confirmation by mass-balance analysis.

The differences among the extruded treatments indicate changes in the measured composition across the selected samples. Because neither mass balance nor protein solubility was determined, the total-protein results are reported descriptively and are not used to infer a specific protein molecular mechanism.

#### 3.2.2. Scanning Electron Microscopy (SEM) Analysis

**Figure 2:**
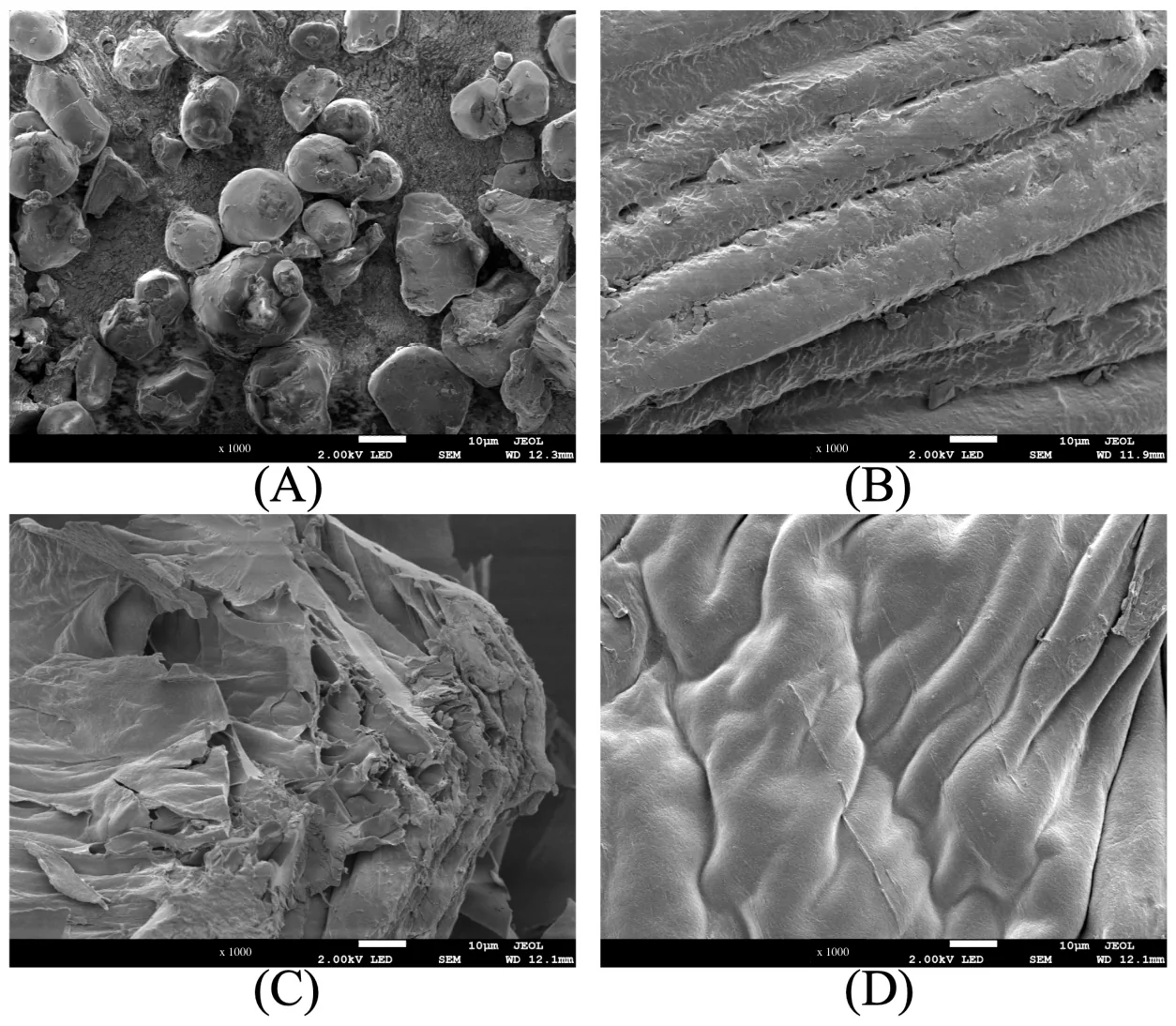
SEM micrographs of native and extruded yellow waxy corn flour: (**A**) YC-YL; (**B**) YC-T180M12; (**C**) YC-T180M15; and (**D**) YC-T180M18.

Figure 2 illustrates the morphological changes in yellow waxy corn flour before and after extrusion processing. As shown in Figure 2A, the native sample (YC-YL) consisted of intact starch granules with smooth surfaces and well-defined polygonal or oval shapes. The granules exhibited a relatively uniform size distribution and remained discrete, without obvious surface defects, reflecting the typical morphology of native waxy corn starch [25].

In contrast, the original granular structure completely disappeared after extrusion (Figure 2B–D). The extruded samples exhibited irregular flaky and lamellar fragments with rough surfaces, accompanied by numerous pores, fissures, and collapsed regions. These morphological changes are attributed to the combined effects of heat, pressure, and mechanical shear during extrusion, which disrupted the integrity of starch granules and promoted the formation of a continuous porous matrix. Similar structural disruption has been widely reported in extruded cereal starches and is considered a characteristic feature of extensive starch gelatinization and matrix expansion [26].

Differences in feed moisture also influenced the surface morphology of the extruded flour samples. Compared with YC-T180M12, the YC-T180M18 sample exhibited a relatively more continuous and homogeneous sheet-like structure with fewer large fractures, suggesting that higher feed moisture enhanced starch plasticization during extrusion and facilitated the formation of a more uniform matrix. Nevertheless, abundant micropores remained distributed throughout the extruded particles, providing numerous channels for rapid water penetration [27].

The formation of a porous and rough microstructure is beneficial for the instant properties of extruded yellow waxy corn flour. Compared with the intact native granules, the porous matrix increases the contact area between particles and water, thereby accelerating water infiltration and particle hydration during reconstitution [28]. These morphological characteristics are consistent with the improved dispersibility, reduced tendency to cake, and enhanced reconstitution performance observed in the extrusion-treated samples.

#### 3.2.3. Particle Size Distribution

The particle size distribution and specific surface area of native and extruded yellow waxy corn flour samples are summarized in Table 4. Native yellow waxy corn flour (YC-YL) exhibited a median particle size (D_50_) of 235.7 μm. After extrusion and subsequent milling through a 60-mesh sieve, the D_50_ values increased significantly to 398.6–452.1 μm (*p* < 0.05). This increase can be attributed to the formation of expanded porous extrudates during extrusion, which generated larger and less dense particles than the native starch granules [29]. Similar increases in particle size have been widely reported for extruded cereal powders owing to starch gelatinization, matrix expansion, and particle restructuring under thermomechanical treatment [30].

Consistent with the increase in particle size, the specific surface area of the extruded samples decreased significantly from 19.58 to 4.65–5.87 m^2^/kg. The larger particle size resulted in a lower external surface area per unit mass, reflecting the structural transformation from compact native granules to expanded porous extrudates [31]. Although the geometric specific surface area decreased, the porous internal structure observed by SEM likely facilitated rapid water penetration into the particles during reconstitution [32].

Among the extruded samples, YC-T180M15 exhibited the smallest D_50_ (398.6 μm) and the highest specific surface area, whereas YC-T180M18 showed the largest particle size and the lowest specific surface area. These differences indicate that feed moisture influenced the expansion behavior and structural compactness of the extrudates during extrusion. Overall, the formation of larger porous particles reduced the number of particle–particle contact points while maintaining efficient water penetration through the internal pore network, thereby contributing to the improved dispersibility and reduced caking tendency of the extruded yellow waxy corn flour samples.

#### 3.2.4. X-Ray Diffraction (XRD) Analysis

**Figure 3:**
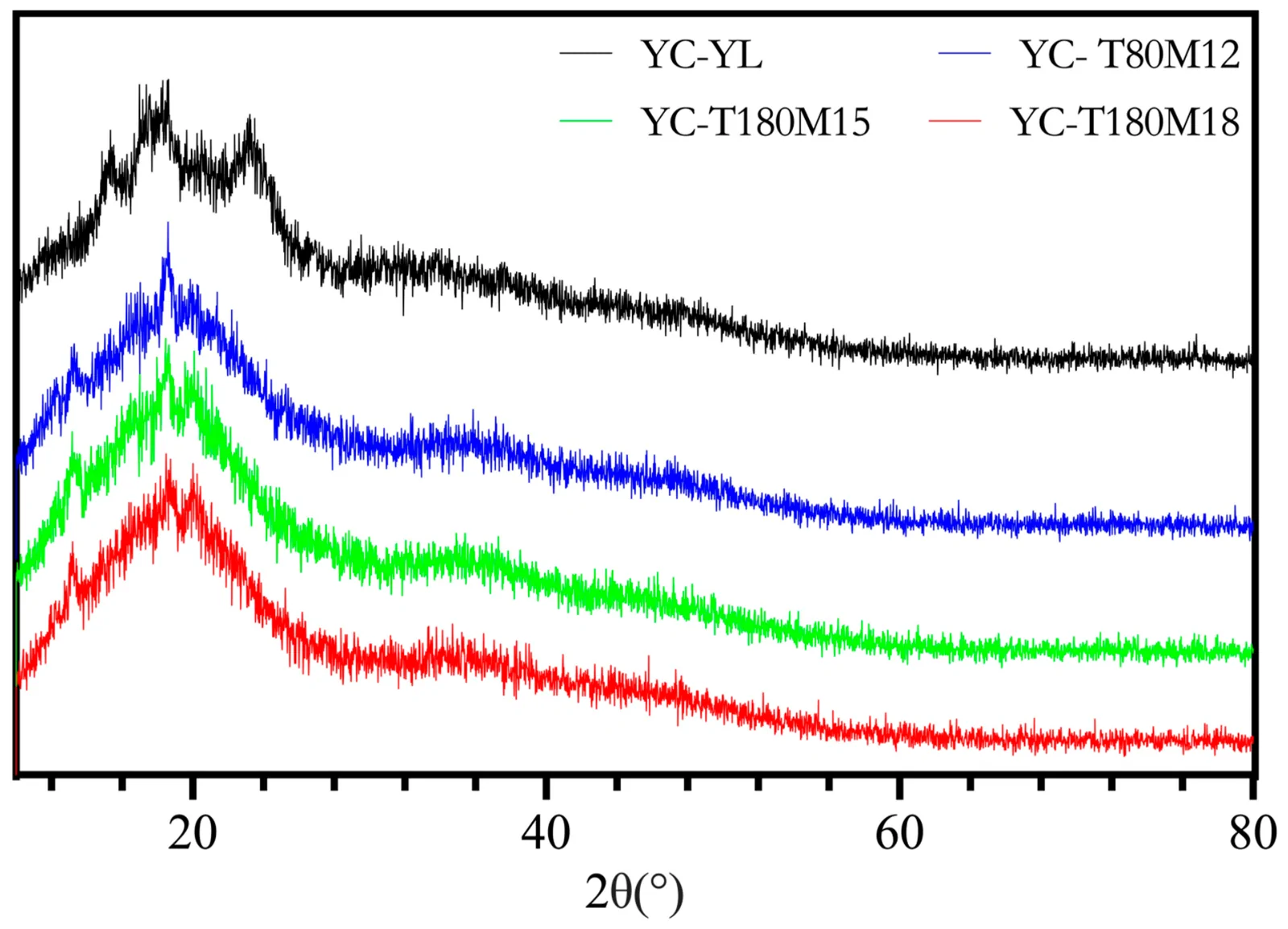
X-ray diffraction patterns of native and extruded yellow waxy corn flour.

Figure 3 presents the X-ray diffraction patterns of native yellow waxy corn flour (YC-YL) and the representative extruded samples. The native sample exhibited characteristic diffraction peaks at approximately 15°, 17°, 18°, and 23° (2θ), with the typical doublet at 17° and 18°, confirming the A-type crystalline structure of waxy corn starch.

The extruded samples retained the same characteristic diffraction peak positions as the native flour, indicating that extrusion under the present processing conditions did not induce a transition in starch crystalline polymorphism [33]. Although extrusion substantially altered the granular morphology, as observed by SEM, the residual crystalline regions maintained the original A-type crystal arrangement.

The relative crystallinity of YC-YL, YC-T180M12, YC-T180M15, and YC-T180M18 was 35.83%, 35.21%, 36.40%, and 36.11%, respectively. No significant differences were observed among the samples, suggesting that extrusion at 180 °C combined with different feed moisture levels exerted only a limited influence on the overall crystalline fraction of the starch. This result indicates that the thermomechanical treatment mainly disrupted the physical integrity of starch granules rather than completely destroying their crystalline organization.

#### 3.2.5. Fourier Transform Infrared Spectroscopy (FTIR) Analysis

**Figure 4:**
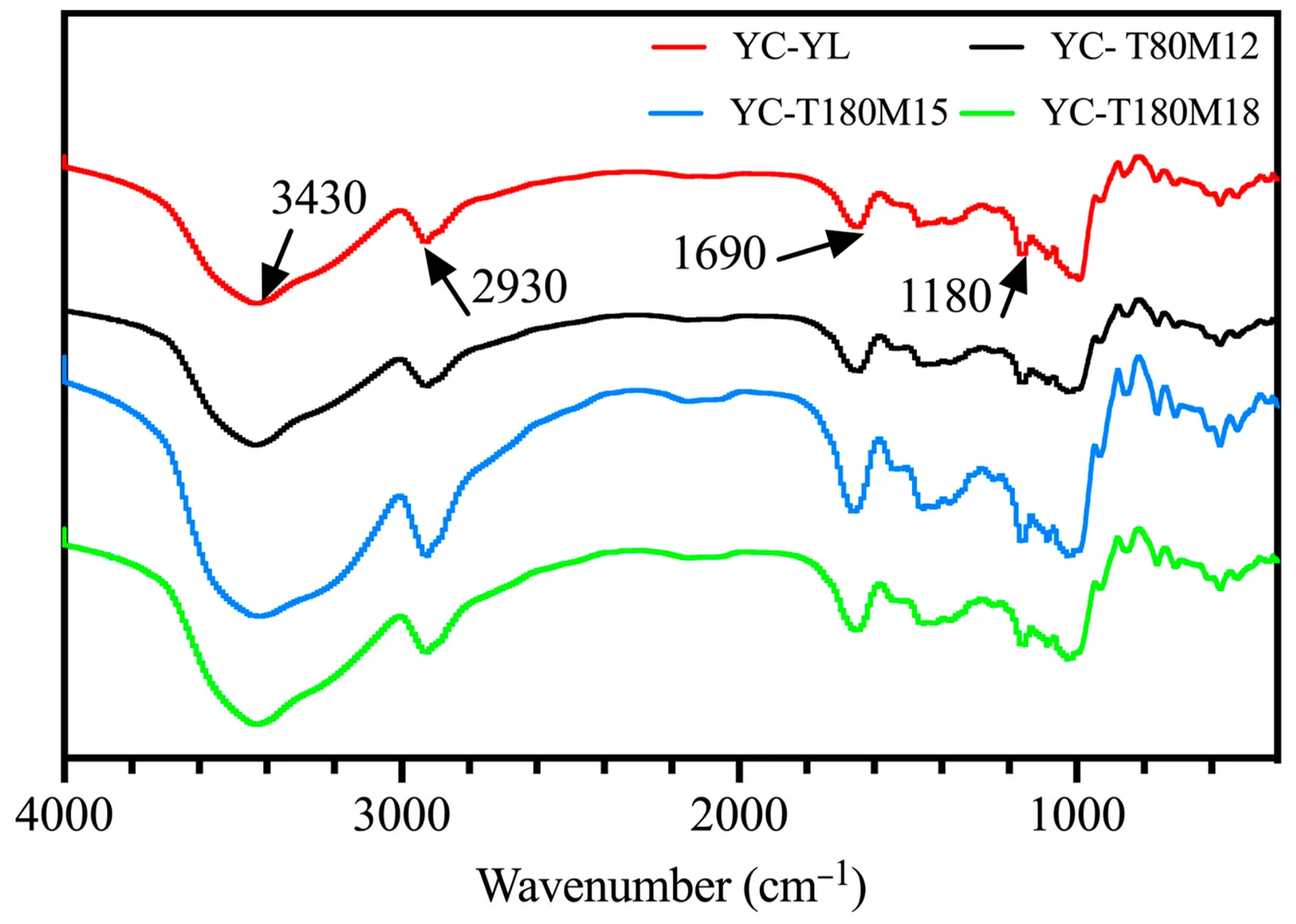
FTIR spectra of native and extruded yellow waxy corn flour.

Figure 4 presents the FTIR spectra of native yellow waxy corn flour (YC-YL) and the representative extruded samples (YC-T180M12, YC-T180M15, and YC-T180M18). All samples exhibited the principal absorption bands at approximately 3430, 2930, 1690, 1180, and 1022 cm^−1^, without the appearance of new prominent bands after extrusion. This indicates that extrusion altered the molecular environment of the flour but did not generate readily detectable new major functional groups.

Although the overall spectral profiles were similar, discernible sample-dependent differences were observed. Compared with YC-YL and YC-T180M12, YC-T180M15 and YC-T180M18 exhibited broader and more pronounced O–H stretching bands near 3430 cm^−1^, suggesting greater changes in the hydrogen-bonding environment at higher feed moisture levels. Differences were also evident near 1690 cm^−1^, where the absorption features of YC-T180M15 and YC-T180M18 were more pronounced than those of YC-YL and YC-T180M12. For whole waxy corn flour, these variations may reflect changes in bound-water interactions and the local environment of carbonyl- or amide-related groups.

At approximately 1180 cm^−1^, YC-T180M15 and YC-T180M18 also showed clearer changes in band shape and relative intensity than YC-YL and YC-T180M12, indicating modification of the C–O–C and C–O vibration environment within the polysaccharide framework [34,35]. Variations near 1022 cm^−1^ further suggest changes in local starch-chain organization. Because spectral deconvolution and numerical band-ratio analysis were not performed, these differences were interpreted qualitatively rather than used to establish a quantitative ranking of short-range molecular order. Together with the SEM and DSC results, the FTIR spectra support differences in hydrogen bonding and local molecular organization among the three feed-moisture treatments during extrusion.

#### 3.2.6. Thermal Transition Behavior

**Figure 5:**
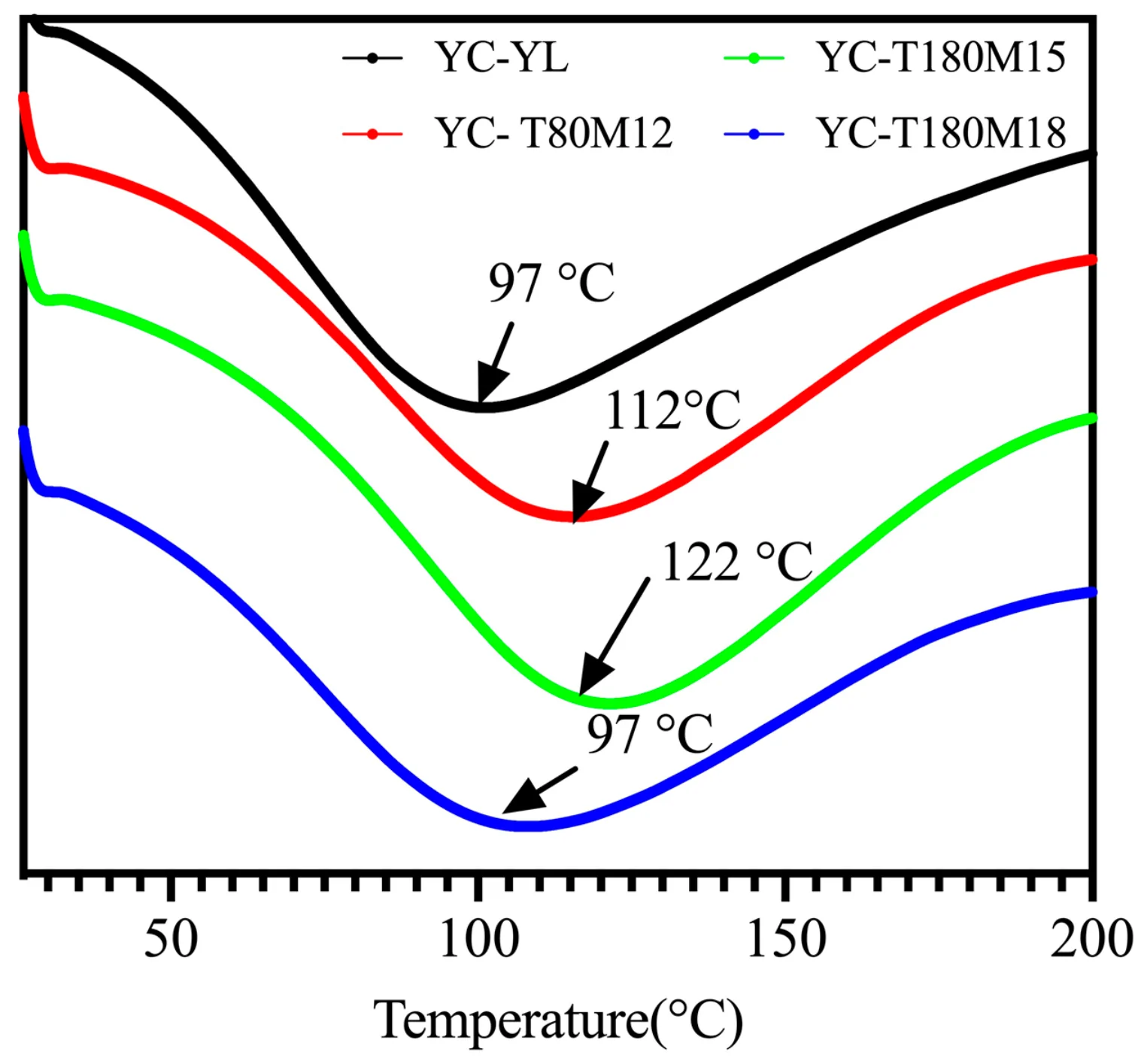
Differential scanning calorimetry (DSC) thermograms of native and extruded yellow waxy corn flour samples.

Figure 5 presents the DSC thermograms of native yellow waxy corn flour (YC-YL) and the representative extruded samples. Native YC-YL exhibited a distinct gelatinization endothermic peak at approximately 97 °C with a gelatinization enthalpy (ΔH) of 8.54 J g^−1^. After extrusion, the ΔH values of all samples decreased markedly to approximately 1.0 J g^−1^, indicating that extrusion substantially reduced the residual ordered structure of starch [36]. Meanwhile, the peak temperature shifted from 97 °C for YC-YL to 112 °C and 122 °C for YC-T180M12 and YC-T180M15, respectively, whereas YC-T180M18 exhibited a lower transition temperature close to that of the native flour.

The pronounced reduction in ΔH indicates that much of the starch had undergone gelatinization or loss of thermal order during extrusion and therefore required less energy for the subsequent transition [37,38]. The higher transition temperatures of YC-T180M12 and YC-T180M15, together with the lower value of YC-T180M18, show that feed moisture altered the residual thermally responsive domains; however, DSC alone cannot identify the molecular associations responsible for these shifts. Similar variations in thermal responses across feed-moisture treatments have been reported in extruded flours and reflect the balance among plasticization, gelatinization, and molecular rearrangement [39,40,41]. The DSC evidence is consistent with the disrupted morphology observed by SEM, whereas FTIR showed broadly preserved functional-group profiles rather than a clearly resolved ranking of molecular order.

### 3.3. Functional Properties of Representative Extruded Flour Samples

#### 3.3.1. Water Absorption Behavior

The water absorption index (WAI) of native and extruded yellow waxy corn flour samples is presented in Table 5. Native yellow waxy corn flour (YC-YL) exhibited a WAI of 0.62 ± 0.03, whereas extrusion significantly increased the WAI to 4.24–7.45 (*p* < 0.05), representing an approximately 7–12-fold increase in water absorption capacity. Among the extruded samples, WAI increased progressively with feed moisture, reaching the highest value in YC-T180M18 (7.45 ± 0.23). These results indicate that increasing feed moisture during extrusion markedly enhanced the hydration capacity of yellow waxy corn flour.

The large WAI increase is consistent with extrusion-induced disruption of native granules and formation of a porous, pregelatinized matrix. The resulting exposure of hydrophilic sites and internal pore channels can increase water binding. The progressive increase from YC-T180M12 to YC-T180M18 suggests that, at 180 °C, greater feed moisture favored water-holding structures despite its potential to reduce mechanical shear. Similar studies have reported that WAI may increase or decrease with temperature and moisture depending on the balance between gelatinization, dextrinization, and matrix fragmentation [11,41]. Thus, the present trend should be regarded as specific to the tested waxy corn matrix and process window, rather than a universal effect of moisture.

The WAI results agree most directly with the porous morphology observed by SEM and the reduced residual gelatinization enthalpy measured by DSC. Together, these findings support easier water penetration and binding after extrusion. Because FTIR differences were small, the hydration improvement is not attributed to a quantitatively demonstrated change in short-range molecular order. Within the selected series, the high WAI of YC-T180M18 helps explain its low caking value, although its supernatant separation ratio shows that water absorption alone does not determine every aspect of reconstitution performance.

#### 3.3.2. Rheological Properties

**Figure 6:**
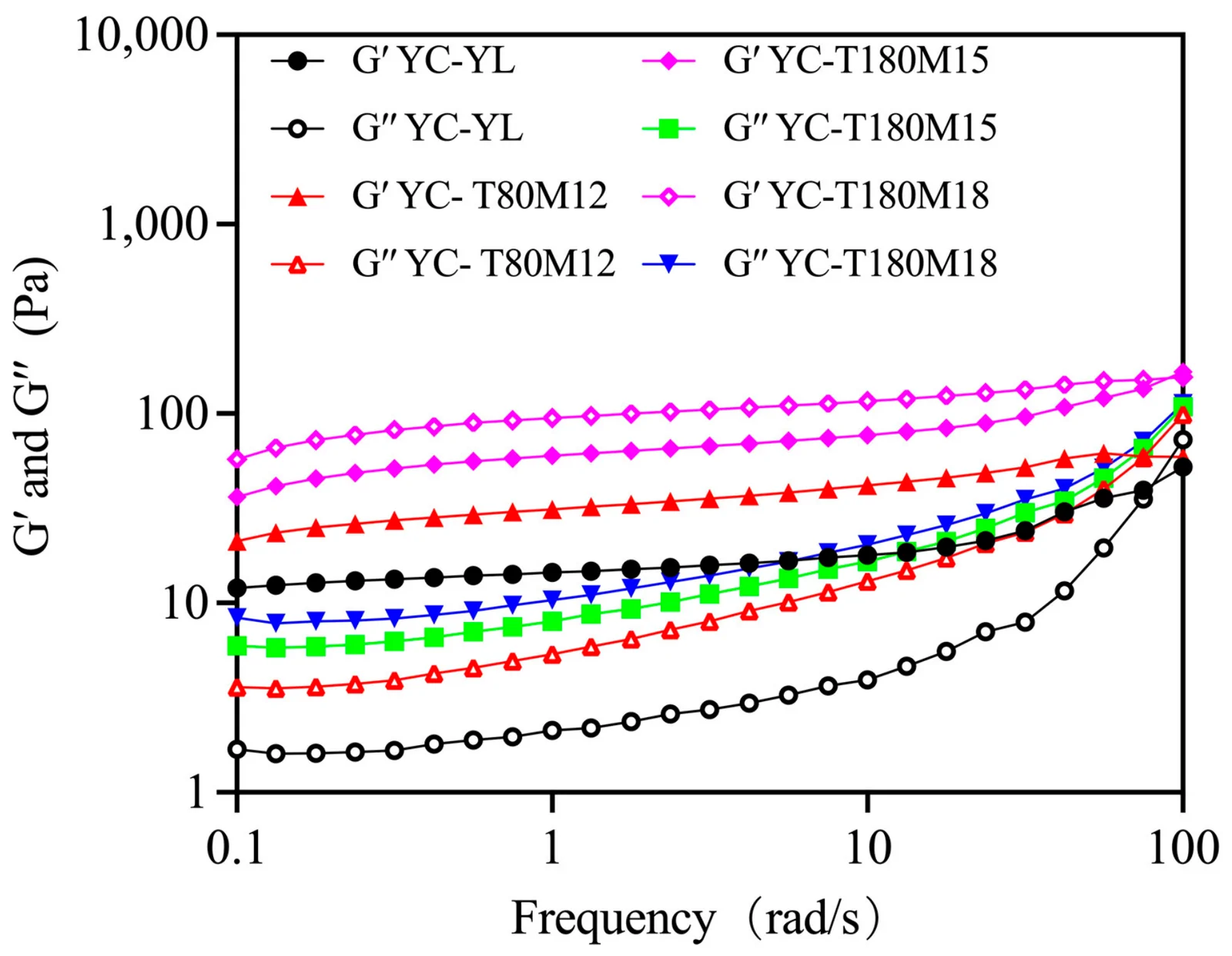
Frequency dependence of the storage modulus (G′) and loss modulus (G″) of reconstituted native and extruded yellow waxy corn flour samples.

Figure 6 presents the frequency-dependent viscoelastic behavior of native yellow waxy corn paste and the representative extruded samples. Both the storage modulus (G′) and loss modulus (G″) increased gradually with increasing frequency, indicating typical frequency-dependent viscoelastic behavior. Throughout the tested frequency range, G″ remained higher than G′, suggesting that all samples behaved predominantly as viscous systems with weak gel characteristics.

Compared with native flour, all extruded samples exhibited higher G′ and G″ values, indicating stronger viscoelastic responses after reconstitution. This result is consistent with the much higher WAI and suggests that the extruded matrices formed more concentrated hydrated networks. Recent work on extruded starch–protein systems similarly links rheological changes to extrusion-induced molecular rearrangement and component interactions [41,42]. However, the relatively small separation among the three extruded curves indicates that rheology was less sensitive to feed moisture than WAI within the selected 180 °C series. Because molecular-weight distribution and starch–protein interactions were not measured, the proposed network mechanism remains an interpretation rather than direct evidence.

The improved viscoelasticity is consistent with the enhanced water absorption capacity and porous microstructure observed after extrusion, which together contributed to the improved suspension stability and reconstitution performance of the instant yellow waxy corn flour samples.

#### 3.3.3. Color Characteristics

The color parameters of native and extruded yellow waxy corn flour samples are summarized in Table 6. Compared with the YC-YL, extrusion significantly altered the color characteristics of the samples (*p* < 0.05). Specifically, the L* value decreased from 91.03 for YC-YL to 85.57 for the extruded flour samples, indicating a reduction in brightness. Meanwhile, the a* value increased markedly from −8.50 to 2.47, representing a pronounced shift from a greenish tone toward a reddish hue. The b* value also decreased from 40.27 to 36.08, suggesting a slight weakening of the characteristic yellow color of yellow waxy corn.

**Figure 7:**
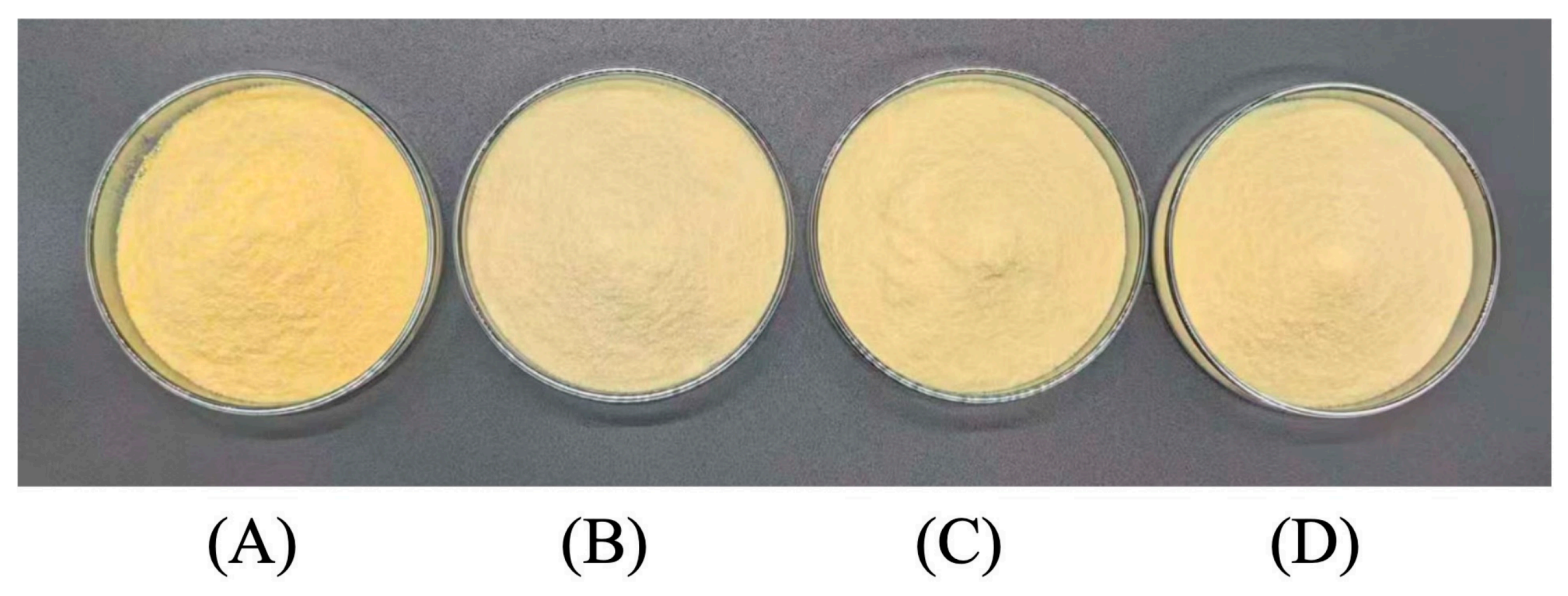
Representative appearance of native and extruded yellow waxy corn flour samples. (**A**) YC-YL; (**B**) YC-T180M12; (**C**) YC-T180M15; (**D**) YC-T180M18.

As shown in Figure 7, the unextruded yellow waxy corn flour (Figure 7A) exhibited a more vivid yellow appearance, whereas the three extruded samples (Figure 7B–D) appeared less intensely yellow and were visually similar to one another. This visual observation was generally consistent with the instrumental color measurements. The color shift may be associated with non-enzymatic browning, caramelization, pigment degradation, or their combined effects during extrusion [43]. Maillard reactions are a plausible contributor because reducing sugars and amino compounds were present in the raw material; however, no Maillard-specific marker was measured. The decreases in L* and b* and the increase in a* therefore demonstrate overall thermal color development but do not identify a unique chemical mechanism.

No significant differences in L* or b* were observed among the three extruded samples, and a* decreased only slightly as feed moisture increased. Thus, within the tested 180 °C series, feed moisture had a smaller effect on color than extrusion itself. The moderate overall change suggests that reconstitution was improved without eliminating the characteristic yellow appearance, although consumer acceptance must be verified by sensory testing.

## 4. Conclusions

Twin-screw extrusion, combined with control of the milled particle-size fraction, improved the hot-water reconstitution of yellow waxy corn flour. The key scientific contribution is the process–structure–function link established for a whole waxy corn matrix: the 60-mesh extruded samples combined low caking with acceptable suspension behavior, while SEM, DSC, and WAI showed that granular disruption, porous-matrix formation, reduced residual gelatinization enthalpy, and enhanced water binding accompanied the improved reconstitution response. XRD and FTIR further indicated retention of the A-type diffraction pattern and the absence of readily detectable new major functional groups. These findings support extrusion as a clean-label route for developing instant porridge powders, while also showing non-monotonic variation among the tested treatment groups. The 180 °C treatments were representative screening selections rather than statistically optimized conditions. The study is limited by the absence of specific mechanical energy and residence-time measurements, quantitative FTIR band analysis, starch digestibility and nutrient-bioavailability data, Maillard-specific markers, sensory evaluation, storage stability, and pilot- or industrial-scale validation. Future work should apply response-surface or multi-objective optimization with independent validation, quantify gelatinization and molecular degradation, assess in vitro digestibility and nutritional quality, evaluate sensory acceptance and shelf-life, and confirm process robustness and energy requirements at industrial scale.

## Figures and Tables

**Table 1:** Caking rate (Q) of 60-mesh yellow waxy corn flour treated under various extrusion barrel temperatures and feed moisture levels.

Samples	Temperature (°C)	Feed Moisture (%)	Q (%)
YC-T140M12	140	12	0.40
YC-T140M15		15	2.66
YC-T140M18		18	1.20
YC-T160M12	160	12	0.16
YC-T160M15		15	0.14
YC-T160M18		18	0.15
YC-T180M12	180	12	0.60
YC-T180M15		15	0.27
YC-T180M18		18	0.13

**Table 2:** Suspension stability coefficient (K) of 60-mesh extruded yellow waxy corn flour under different thermal-shear treatments.

Samples	Temperature (°C)	Feed Moisture (%)	K (%)
YC-T140M12	140	12%	8.00
YC-T140M15		15%	20.00
YC-T140M18		18%	15.00
YC-T160M12	160	12%	8.00
YC-T160M15		15%	10.00
YC-T160M18		18%	11.00
YC-T180M12	180	12%	8.00
YC-T180M15		15%	4.00
YC-T180M18		18%	11.00

**Table 3:** Total starch, reducing sugar, and total protein contents of native and extruded yellow waxy corn flour.

Samples	Starch (%)	Reducing Sugar (g/100 g)	Total Protein (%)
YC-YL	73.0 ± 0.5 ^a^	1.0 ± 0.0 ^a^	0.10 ± 0.00 ^b^
YC-T180M120M	73.1 ± 0.8 ^b^	ND	0.56 ± 0.02 ^a^
YC-T180M150M	73.4 ± 0.6 ^a^	ND	0.63 ± 0.03 ^a^
YC-T180M18	75.3 ± 0.7 ^a^	ND	0.66 ± 0.01 ^a^

Note: Values within the same column followed by different lowercase superscript letters are significantly different (*p* < 0.05); ND = not detected (content below instrument detection limit).

**Table 4:** Particle-size distribution and specific surface area of native and extruded yellow waxy corn flour.

Samples	D_50_ (μm)	Specific Surface Area (m^2^/kg)
YC-YL	235.7 ± 3.6 ^c^	19.58 ± 0.52 ^a^
YC-T180M12	425.3 ± 12.5 ^a^	5.23 ± 0.31 ^b^
YC-T180M15	398.6 ± 10.2 ^b^	5.87 ± 0.28 ^b^
YC-T180M18	452.1 ± 15.8 ^a^	4.65 ± 0.35 ^b^

Note: Different lowercase letters in the same column indicate significant differences between groups (*p* < 0.05).

**Table 5:** Water absorption index (WAI) of native and extruded yellow waxy corn flour samples.

Samples	WAI (g/g)
YC-YL	0.62 ± 0.03 ^a^
YC-T180M12	4.24 ± 0.21 ^b^
YC-T180M15	4.74 ± 0.13 ^c^
YC-T180M18	7.45 ± 0.23 ^d^

Note: Different lowercase letters in the same column indicate significant differences between groups (*p* < 0.05).

**Table 6:** Color parameters of native and extruded yellow waxy corn flour samples.

Samples	L*	a*	b*
YC-YL	91.03 ± 0.233^a^	−8 ± 0.70 ^b^	40.27 ± 0.78 ^a^
YC-T180M120M	86.47 ± 2.63 ^b^	3.23 ± 0.23 ^a^	36.23 ± 0.87 ^b^
YC-T180M15	85.58 ± 0.38 ^b^	2.68 ± 0.36 ^a^	36.08 ± 1.15 ^b^
YC-T180M18	85.57 ± 0.21 ^b^	2.47 ± 0.06 ^a^	37.43 ± 0.21 ^b^

Note: Different lowercase letters in the same column indicate significant differences between groups (*p* < 0.05).

## Data Availability

The original contributions presented in this study are included in the article/Supplementary Materials. Further inquiries can be directed to the corresponding author.

## Supplementary Materials

The following supporting information can be downloaded at: https://www.mdpi.com/article/10.3390/foods15172974/s1, Table S1. Caking rate (Q) of yellow waxy corn flour with different particle sizes treated under various extrusion barrel temperatures and feed moisture levels; Table S2. Suspension stability coefficient (K) of yellow waxy corn flour with different particle sizes treated under various extrusion barrel temperatures and feed moisture levels.
