# Supplementary material for: Effect of Extrusion Processing on the Instant Properties and Structural Characteristics of Yellow Waxy Corn Flour
Source: Foods. 2026 Aug 25;15(17):2974. doi: 10.3390/foods15172974 (PMC13565714; doi:10.3390/foods15172974)
Supplement: Supplementary file 1 [file foods-15-02974-s001.zip › foods-4488189-supplementary.pdf]

Effect of Extrusion Processing on the Instant Properties and Structural Characteristics of Yellow Waxy Corn Flour

Hong Guo [1], Zhongdong Zhang [2], Zhe Cheng [1], Qi Li [1] and Yunlong Li [1,*]

[1] Shanxi Institute for Functional Food, Shanxi Agricultural University, Taiyuan 030031, China
[2] Maize Research Institute, Shanxi Agricultural University, Xinzhou 034000, China
* Correspondence: liyunlong125@126.com

Table S1. Caking rate (Q) of yellow waxy corn flour with different particle sizes treated under various extrusion barrel temperatures and feed moisture levels

Samples	Temperature (℃)	Feed Moisture (%)	Different particle sizes (mesh)					
			40	45	50	60	70	80
1	140℃	12%	0.12	0.05	0.03	0.40	0.57	8.8
2		15%	4.60	3.27	2.61	2.66	6.29	11.0
3		18%	4.38	3.18	1.68	1.20	2.24	5.15
4	160℃	12%	0.38	0.33	0.21	0.16	0.43	1.22
5		15%	0.77	0.49	0.25	0.14	0.39	3.77
6		18%	0.71	0.33	0.21	0.15	0.23	1.20
7	180℃	12%	0.74	0.56	0.32	0.60	0.76	5.01
8		15%	0.41	0.23	0.11	0.27	0.80	2.79
9		18%	0.64	0.33	0.19	0.13	0.16	1.08

Table S2. Suspension stability coefficient (K) of yellow waxy corn flour with different particle sizes treated under various extrusion barrel temperatures and feed moisture levels

Samples	Temperature (℃)	Feed Moisture (%)	Different particle sizes (mesh)					
			40	45	50	60	70	80
1	140℃	12%	0.44	0.30	0.20	0.08	0.04	0.04
2		15%	0.79	0.68	0.38	0.20	0.04	0.04
3		18%	1.20	0.78	0.33	0.15	0.04	0.04
4	160℃	12%	0.28	0.27	0.15	0.08	0.08	0.04
5		15%	0.58	0.44	0.21	0.10	0.04	0.04
6		18%	0.46	0.42	0.18	0.11	0.04	0.04
7	180℃	12%	0.34	0.33	0.11	0.08	0.04	0.04
8		15%	0.49	0.38	0.21	0.04	0.04	0.04
9		18%	0.43	0.37	0.17	0.11	0.04	0.04
